# Dasatinib Ointment Promotes Healing of Murine Excisional Skin Wound

**DOI:** 10.1021/acsptsci.2c00245

**Published:** 2023-06-09

**Authors:** Surasak Wichaiyo, Saovaros Svasti, Arnatchai Maiuthed, Pattarawit Rukthong, Arman Syah Goli, Noppawan Phumala Morales

**Affiliations:** † Department of Pharmacology, Faculty of Pharmacy, Mahidol University, Bangkok 10400, Thailand; ‡ Centre of Biopharmaceutical Science for Healthy Ageing, Faculty of Pharmacy, Mahidol University, Bangkok 10400, Thailand; § Thalassemia Research Center, Institute of Molecular Biosciences, Mahidol University, Nakhon Pathom 73170, Thailand; ∥ Department of Biochemistry, Faculty of Science, Mahidol University, Bangkok 10400, Thailand; ⊥ Department of Pharmaceutical Technology, Faculty of Pharmacy, Srinakharinwirot University, Nakhonnayok 26120, Thailand; # Department of Pharmacology, Faculty of Science, Mahidol University, Bangkok 10400, Thailand

**Keywords:** Dasatinib ointment, inflammation, vascular integrity, wound healing

## Abstract

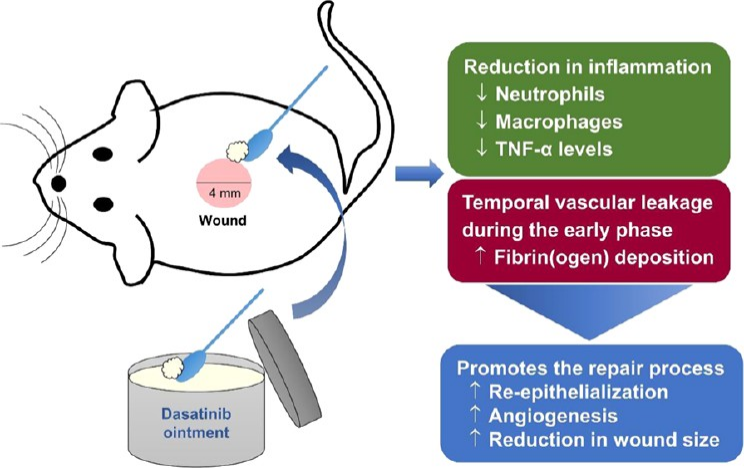

Dasatinib, a tyrosine kinase inhibitor, has been shown to produce anti-inflammatory activity and impair vascular integrity in vivo, including during skin wound healing, potentially promoting the repair process. Given that dasatinib is a lipophilic small molecule capable of penetrating skin, topical dasatinib might provide benefits in wound healing. In the present study, we investigated the impact of dasatinib ointments in skin wound healing in mice. A full thickness excisional skin wound (4 mm diameter) was generated on the shaved dorsum of eight-week-old C57BL/6 mice. Dasatinib ointment (0.1 or 0.2% w/w) or ointment base was applied twice daily (every 12 h) for 10 days. Elizabethan collars were used to prevent animal licking. The wound size was monitored daily for 14 days. The results showed that dasatinib ointments, particularly 0.1% dasatinib, promoted a 16–23% reduction in wound size (*p* < 0.05) during day 2 to day 6 postinjury compared to controls. Immunohistochemistry analyses demonstrated a reduction in wound neutrophils (38% reduction, *p* = 0.04), macrophages (47% reduction, *p* = 0.005), and tumor necrosis factor-α levels (73% reduction, *p* < 0.01), together with an induction of vascular leakage-mediated fibrin(ogen) accumulation (2.5-fold increase, *p* < 0.01) in the wound during day 3 postinjury (an early phase of repair) in 0.1% dasatinib-treated mice relative to control mice. The anti-inflammatory and vascular hyperpermeability activities of dasatinib were associated with an enhanced healing process, including increased keratinocyte proliferation (1.8-fold increase in Ki67^+^ cells, *p* < 0.05) and augmented angiogenesis (1.7-fold increase in CD31^+^ area, *p* < 0.05), compared to the ointment base-treated group. Following treatment with 0.2% dasatinib ointment, minor wound bleeding and scab reformation were observed during the late phase, which contributed to delayed healing. In conclusion, our data suggest that dasatinib ointment, mainly at 0.1%, promotes the repair process by reducing inflammation and producing a local and temporal vascular leakage, leading to an increase in fibrin(ogen) deposition, re-epithelialization, and angiogenesis. Therefore, topical dasatinib might be a potential novel candidate to facilitate skin wound healing.

Wound healing is a complex pathobiological mechanism, comprised of hemostasis, inflammation, proliferation, and remodeling phases.^1^ It has been suggested that accelerated wound healing might be promoted by several therapeutic strategies, including attenuation of inflammation, activation of keratinocyte proliferation and migration, and promotion of angiogenesis.^2−4^ Increased vascular leakage has also been shown to facilitate cutaneous wound healing by promoting extravasation of plasma-derived proteins, including fibrinogen and growth factors (such as platelet-derived growth factor, fibroblast growth factor, and transforming growth factor), which contribute to the repair process.^5−8^ For example, histamine^9^ or the serum fraction of the natural latex from the rubber tree^10^ increases vascular permeability and accelerates wound healing. In addition, microneedling generates a local and temporal bleeding,^7^ which may promote angiogenesis during the repair process.^7,8^ Given that platelet glycoprotein VI (GPVI) and C-type lectin-like receptor 2 (CLEC-2) maintain vascular integrity to prevent inflammatory bleeding following leukocyte diapedesis,^11^ we have previously shown that targeting these receptors promotes skin wound healing in mice by inducing vascular leakage and reducing inflammation during the inflammatory phase of the repair process (day 1–3 postinjury).^6^ In agreement with this, endothelial pores are approximately 4 μm in width and 6 μm in length during leukocyte diapedesis,^12^ which potentially allows free extravasation of red blood cells^11,13^ (5–6 μm in diameter),^14^ but not neutrophils (∼14 μm in diameter)^15^ and monocytes (12–16 μm in diameter).^15^

Dasatinib is a tyrosine kinase inhibitor indicated for the treatment of chronic myeloid leukemia. It inhibits the breakpoint cluster region-Abelson tyrosine kinase (BCR-ABL) fusion protein and other tyrosine kinases, including Src, Syk, Tec, and cKIT.^16,17^ It has been shown that a low dose of dasatinib (≤10 mg/kg) intraperitoneal (i.p.) injection produces anti-inflammatory activity in several animal models of inflammation,^18^ including in the lungs^18,19^ and the skin.^5^ In addition, this dose-range of dasatinib was capable of blocking platelet aggregation upon GPVI and CLEC-2 activation by inhibiting Src and Syk.^16,20,21^ More recently, our previous work demonstrated that 5 mg/kg dasatinib i.p. injection accelerated skin wound healing in association with transient inflammatory bleeding, decreased inflammation, and enhanced re-epithelialization and angiogenesis.^5^ Interestingly, dasatinib is a lipophilic small molecule (molecular weight = 488 D) with an acceptable octanol–water partition coefficient (log *P* = 2.71),^22^ which potentially allows it to penetrate well through the cell membrane.^22,23^ More importantly, a previous study demonstrated that the topical application of dasatinib solution (0.02–0.1%) dose-dependently reduced inflammation in a murine model of allergic contact dermatitis.^24^ Due to the physicochemical properties of dasatinib,^22,23^ the positive impact of i.p. dasatinib in wound repair,^5^ and its previous anti-inflammatory activity following topical administration,^24^ the present study aimed to investigate the impact of dasatinib ointments in skin wound healing in mice. We hypothesize that topical dasatinib may facilitate wound healing by attenuating inflammation and promoting vascular leakage.

## Methods

### Preparation of Dasatinib Ointments

**Figure 1:**
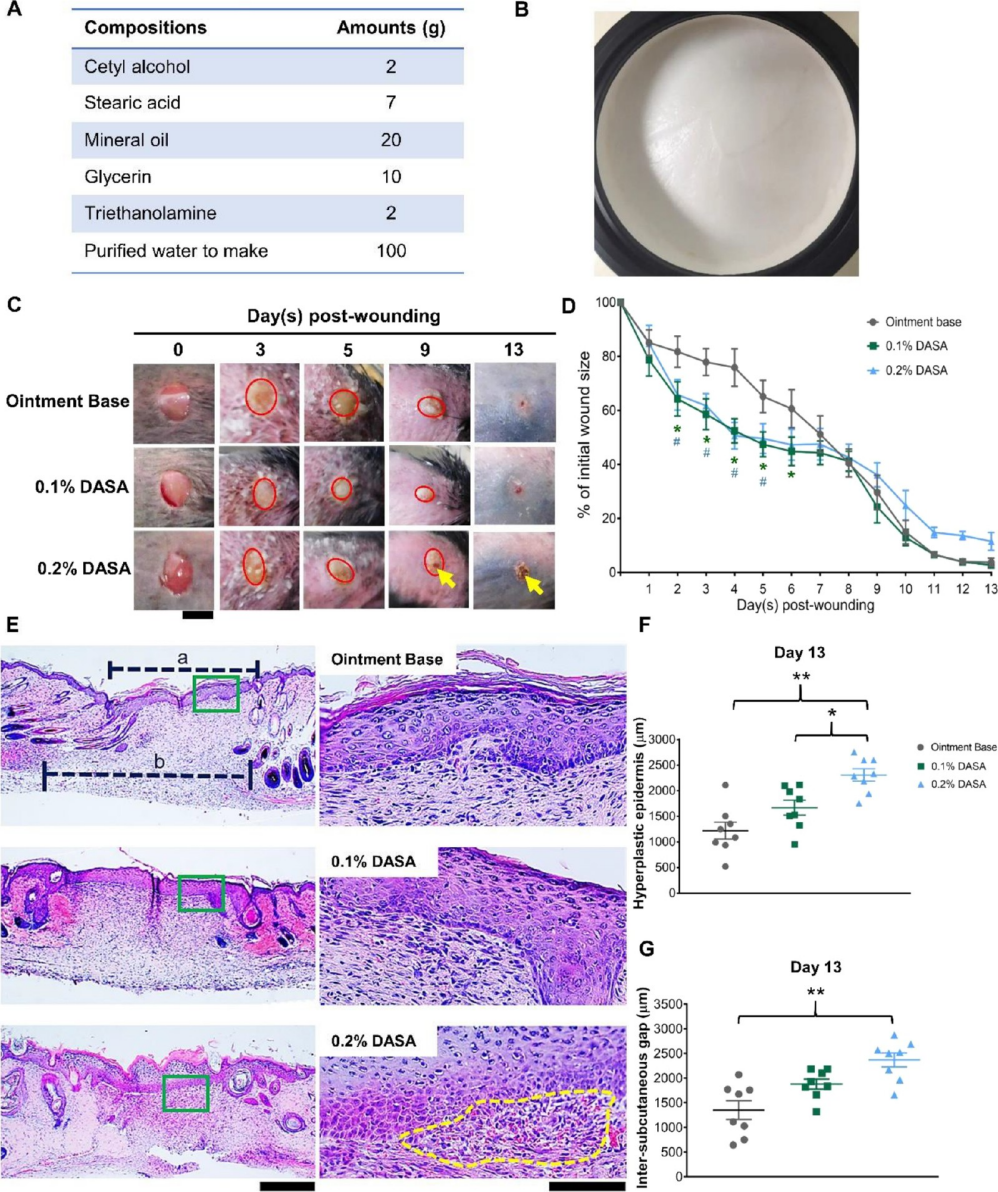
Dasatinib ointment promotes skin wound repair. (A) Composition of ointment base. (B) Texture of ointment. (C) Macroscopic wound appearance over 14 days (*n* = 8). The arrow points to minor wound bleeding and scab reformation. Scale bar = 4 mm. (D) Kinetics of wound closure over 14 days (*n* = 8). 0.1% DASA = 0.1% dasatinib ointment, 0.2% DASA = 0.2% dasatinib ointment. Bars indicate the mean ± SEM. Data are analyzed by two-way ANOVA with Tukey’s multiple comparison test. *p < 0.05 for the comparison between 0.1% DASA and ointment base controls. *#p* < 0.05 for the comparison between 0.2% DASA and ointment base controls. (E) H&E images showing wound scars at day 13 postinjury (*n* = 8). “a” represents the length of the hyperplastic epidermis, “b” shows the inter-subcutaneous gap. The dotted yellow line highlights extravasation of red blood cells in the scar. Magnification = 40× (left) and 400× (right). Scale bar = 500 μm (left) and 100 μm (right). (F) Graph demonstrating the quantification of the length of the hyperplastic epidermis at day 13 postinjury (*n* = 8). (G) Graph presenting the quantification of the distance of the inter-subcutaneous gap at day 13 postinjury (*n* = 8). Bars indicate the mean ± SEM. Data are analyzed by one-way ANOVA with Tukey’s multiple comparison test. *p < 0.05, **p < 0.01.

Topical dasatinib (0.1 and 0.2% w/w) was formulated in a soft water-washable ointment base (Figure 1A,B). The ointment base was prepared as previously described.^25^ To prepare 0.1 and 0.2% ointments, 10 and 20 mg dasatinib powder (SML2589; Sigma) was weighed and added to 10 g ointment, respectively. Dasatinib ointments were freshly prepared for each experiment and kept at 2–8 °C until use.

### Animals

Eight-week-old male and female wild-type (WT) C57BL/6 mice were used. All animal studies were approved by the Faculty of Pharmacy, Mahidol University-Institutional Animal Care and Use Committee under project number PYR 009/2019.

### Skin Wound Model

Mice were divided into three groups: 0.1% dasatinib-treated, 0.2% dasatinib-treated, and ointment base-treated control. To investigate the wound healing activity of the dasatinib ointments, a 4 mm diameter full thickness excisional skin wound was generated on the shaved dorsum of the animals using a biopsy punch (Kai Industries) under isoflurane anesthesia, as previously described.^5,6^ After hemostasis (a few minutes after biopsy), 20–30 mg of dasatinib ointment or ointment base was applied twice daily (every 12 h) for 10 days (day 0 to day 9 postinjury). Elizabethan collars were used to prevent animal licking. Wounds were left open, and the wound size was measured daily for 14 days (day 0 to day 13 postinjury) using calipers. Wound images were taken using a Nikon COOLPIX B500 digital camera.^5,6^ The wound size was calculated and reported as a percentage of the initial size.^5,6^

### Histological Analysis

Wounds at day 3 and day 13 postinjury were collected, fixed in 4% paraformaldehyde, dehydrated, and embedded in paraffin, respectively, using a standard protocol. Skin tissues were cut at 5 μm for histological analyses. Hematoxylin and eosin (H&E) staining was performed, and images were acquired using an Olympus BX63 Motorized Microscope. The histology of the wounds at day 3 postinjury was examined for extravasation of red blood cells. The histology of the wounds at day 13 postinjury was used for microscopic observations and for the measurement of scar size: the length of the hyperplastic epidermis and the inter-subcutaneous gap.^5,6,26^

### Immunohistochemistry

Paraffin-embedded wound sections (5 μm) were used for immunohistochemistry staining. The primary antibodies used in immunohistochemistry were a 1:50 dilution of anti-CD31 polyclonal antibody (ab28364, Abcam) and a 1:200 dilution of the following antibodies: anti-Ki67 polyclonal antibody (ab15580, Abcam), anti-tumor necrosis factor alpha (TNF-α) polyclonal antibody (ab9739; Abcam), anti-Ly6G monoclonal antibody (551459; BD Biosciences), anti-F4/80 monoclonal antibody (MCA497GA; Bio-Rad), anti-CD206 monoclonal antibody (24595; Cell Signaling Technology), and anti-fibrinogen polyclonal antibody (ab27913; Abcam). The secondary antibodies used in this study were a 1:200 dilution of horseradish peroxidase (HRP)-conjugated goat anti-rat IgG (ab97057; Abcam) or HRP-conjugated donkey anti-rabbit IgG (NA934; GE Healthcare). Signal detection was performed using ImpactDAB substrate (SK-4105; Vector Lab).^5,6^

High-power field (HPF) images of the wounds were taken using an Olympus BX63 Motorized Microscope at 400× magnification.^27^ The signal (brown color) was split using the color deconvolution (H-DAB) mode in Fiji software, followed by the threshold (Otsu) setting. The fibrinogen content was measured from the intensity of the signal, which was normalized to area and presented as fold relative to the control. The density of endothelial cells was examined using the area covered by the signal (CD31^+^) per HPF and presented as fold relative to the control. For the quantification of Ki67, Ly6G, F4/80, CD206, and TNF-α, the signal particles with an area of at least 30 square microns were counted and presented per HPF, as previously described.^5,6^

### Enzyme-Linked Immunosorbent Assay (ELISA)

Wound tissues at day 3 postinjury (15 mg) were homogenized in 500 μL of cold phosphate buffer saline supplemented with a protease inhibitor cocktail (5871S; Cell Signaling Technology) and centrifuged at 10,000 × *g* for 20 min at 4 °C.^28^ The supernatants were collected for TNF-α measurement using ELISA kits (MTA00B; R&D Systems). The protein concentration was determined using the Bradford method, and the TNF-α levels were reported per mg protein.

### Platelet Aggregation Assay

Citrated blood was collected via the inferior vena cava under isoflurane anesthesia on day 3 and day 13 postinjury. Platelet-rich plasma was prepared,^21^ half-diluted^29^ with normal saline, and incubated at 37 °C for 3 min. The platelet suspension was then continuously stirred (1200 rpm) at 37 °C for 1 min prior to adding 4 μg/mL collagen (Chrono-Par 385; Chrono-Log) or 0.4 μg/mL rhodocytin (CSB-EP888242CBG; Cusabio Technology).^16,20,21^ Platelet aggregation was measured using a light-transmission aggregometer (Series 490; Chrono-Log).

### Statistical Analysis

Data are presented as mean ± standard error of the mean (SEM). Mean changes in the wound size over 14 days were compared using two-way analysis of variance (ANOVA) with Tukey’s multiple comparison test using GraphPad. For the rest of the parameters, the mean differences were compared using one-way ANOVA with Tukey’s post-hoc analysis or the Kruskal-Wallis test with Dunn’s multiple comparison test. *p* < 0.05 was considered as statistically significant.

## Results

### Dasatinib Ointment Accelerates Skin Wound Healing

To test whether dasatinib ointment affects wound healing, we generated a single (4 mm diameter) excisional wound on the shaved dorsal skin of mice. The ointments were applied twice daily for 10 days (until day 9 postinjury), and wound closure was monitored for 14 days (until day 13 postinjury). The results demonstrated that 0.1% dasatinib ointment did not exhibit any difference in wound appearance (Figure 1C), but significantly accelerated wound closure at day 2 (18% reduction in wound size, *p* < 0.05), with the highest magnification observed at day 4 (23% reduction in wound size, *p* < 0.05), and the effect lasted until day 6 postinjury (16% reduction in wound size, *p* < 0.05), compared to the ointment base-treated controls (Figure 1C,D). There was no difference in wound size following day 7 to day 13 postinjury between these two groups (Figure 1D). In the 0.2% dasatinib ointment-treated mice, wound closure was also promoted at day 2 (16% reduction in wound size, *p* < 0.05), with the highest magnification observed at day 4 (25% reduction in wound size, *p* < 0.05), and the effect lasted until day 5 postinjury (16% reduction in wound size, *p* < 0.05), relative to the ointment base-treated mice (Figure 1D). However, minor wound bleeding was observed at day 9 postinjury, which resulted in unexpected scab reformation at day 10 to day 13 postinjury (Figure 1C). Histological analysis of the scar at day 13 postinjury using H&E staining confirmed the apparent extravasation of red blood cells underneath the hyperplastic epidermis in the 0.2% dasatinib ointment-treated animals but not in the other two groups (Figure 1E). In addition, measurement of the wound scar revealed that mice treated with 0.1% dasatinib had a similar size of scar, including the length of the hyperplastic epidermis, the upper layer (Figure 1F), and the inter-subcutaneous gap, the deeper layer (Figure 1G), as observed in the controls. In contrast, the 0.2% dasatinib-treated mice showed a significantly longer hyperplastic epidermis compared to the other two groups (2308.4 ± 120.9 μm vs 1668.7 ± 145.5 μm in 0.1% dasatinib-treated vs 1219.8 ± 165.4 μm in control, *p* < 0.01) (Figure 1F) and a longer inter-subcutaneous gap relative to the ointment base-treated group (2367.6 ± 142.8 μm vs 1349.9 ± 188.2 μm in control, *p* < 0.01) (Figure 1G) but not compared to the 0.1% dasatinib-treated group (1877.6 ± 103.8 μm).

Taken together, our data demonstrate that a low concentration dasatinib ointment (0.1% w/w), applied twice daily, accelerates skin wound healing, primarily during the early phase, without affecting the late phase of repair in mice. A double dose of dasatinib (0.2% w/w) produces adverse minor bleeding in the later phase, which leads to scab reformation and a larger size of scar at the end.

### Dasatinib Ointment Promotes Keratinocyte Proliferation and Angiogenesis during the Early Phase of Repair

**Figure 2:**
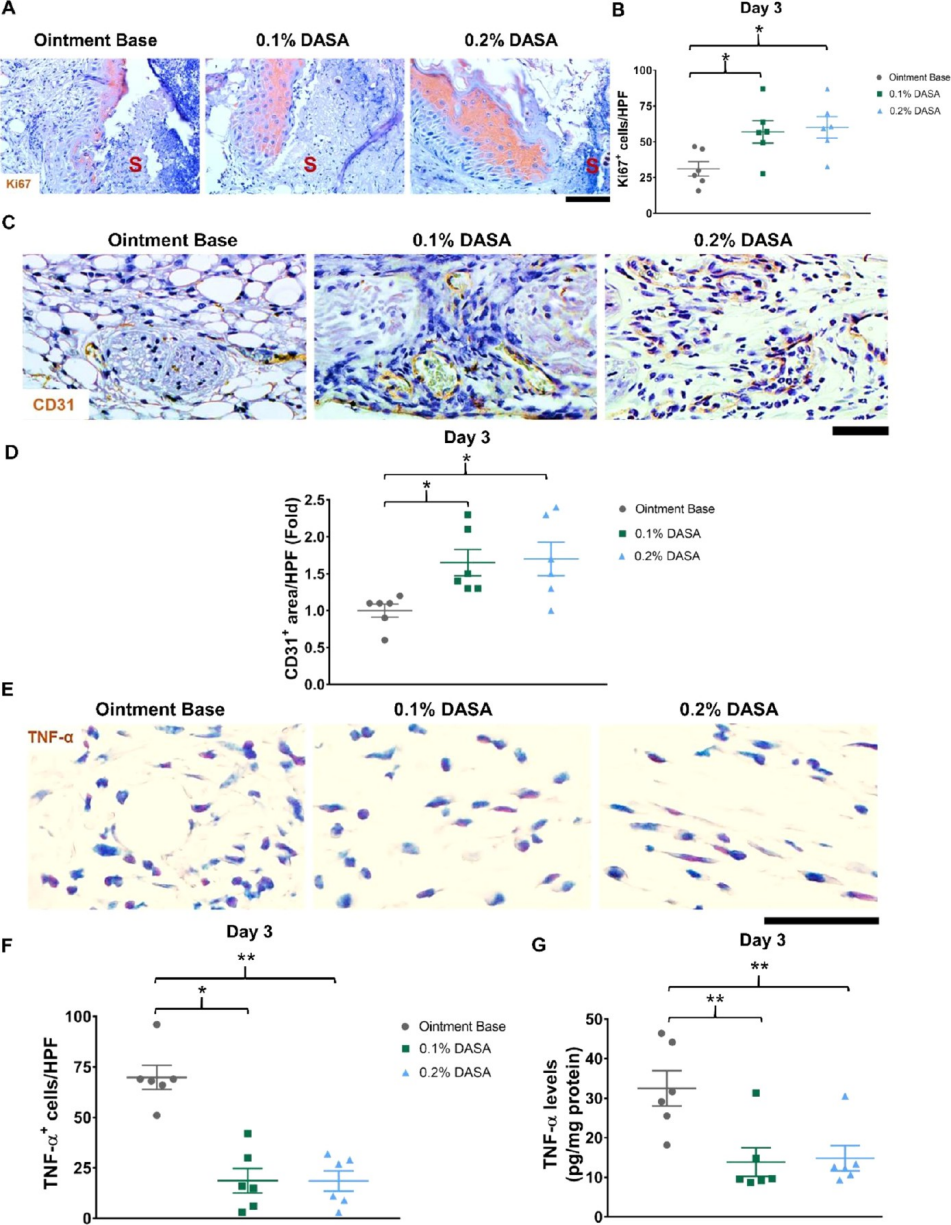
Dasatinib ointment enhances keratinocyte proliferation and angiogenesis but decreases TNF-α levels during the inflammatory phase of wound repair. (A) Immunohistochemistry staining of Ki67 (brown) at day 3 postinjury (*n* = 6). S = scab. (B) Graph showing the relative quantification of Ki67^+^ cells per HPF in the wound at day 3 postinjury (*n* = 6). (C) Immunohistochemistry staining of CD31 (brown) at day 3 postinjury (*n* = 6). (D) Graph presenting the relative quantification of CD31^+^ area per HPF in the wound at day 3 postinjury (*n* = 6). (E) Immunohistochemistry staining of TNF-α (brown) at day 3 postinjury (*n* = 6). (F) Graph demonstrating the relative quantification of TNF-α^+^ cells per HPF in the wound at day 3 postinjury (*n* = 6). (G) Graph showing TNF-α levels in the wound at day 3 postinjury measured using ELISA. Magnification = 400× (A, C, E). Scale bar = 50 μm. 0.1% DASA = 0.1% dasatinib ointment, 0.2% DASA = 0.2% dasatinib ointment. Bars indicate the mean ± SEM. Data are analyzed by one-way ANOVA with Tukey’s multiple comparison test. *p < 0.05, **p < 0.01.

Given that keratinocyte proliferation is activated during re-epithelialization in the repair process,^30−32^ we performed immunohistochemistry staining of Ki67, a marker of cell proliferation, in wound tissues at day 3 postinjury. The data showed that keratinocyte proliferation (Ki67^+^, brown) was mainly observed in the nucleus (Figure S1A) and cytoplasm of keratinocytes at the wound edges (Figures 2A and S1B). Treatment with dasatinib ointment (both 0.1 and 0.2%) significantly increased the numbers of Ki67^+^ keratinocytes compared to the ointment base-treated animals (57 ± 8 cells/HPF in 0.1% dasatinib-treated vs 60 ± 7 cells/HPF in 0.2% dasatinib-treated vs 31 ± 5 cells/HPF in control, *p* < 0.05) (Figure 2B). To investigate the impact of the dasatinib ointments on angiogenesis, immunohistochemistry staining of CD31 (a marker of endothelial cells) was performed. In wound tissues, CD31^+^ cells were primarily detected at the vessel walls (Figure 2C). At day 3 postinjury, the endothelial cell density was significantly increased (1.7-fold increase in CD31^+^ area, *p* < 0.05) in the wounds of mice treated with dasatinib ointments (both 0.1 and 0.2%) compared to the ointment base-treated mice (Figure 2D).

These data indicate that the dasatinib ointments facilitate re-epithelialization and angiogenesis in the early phase, which contributes to the acceleration in wound repair.

### Dasatinib Ointment Reduces Inflammation in Skin Wounds

**Figure 3:**
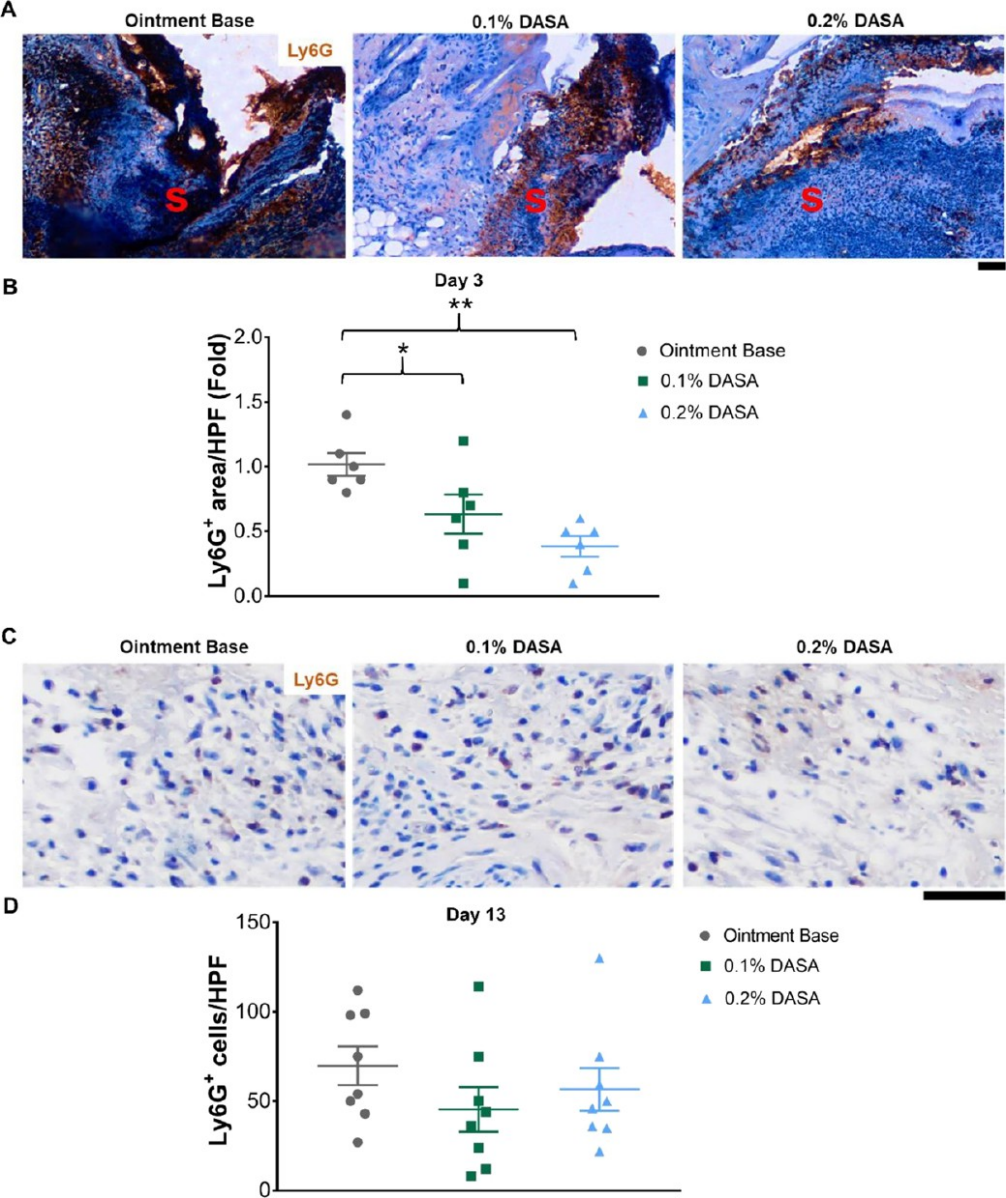
Wound neutrophils are reduced during the inflammatory phase in dasatinib-treated mice. (A) Immunohistochemistry staining of neutrophils (Ly6G^+^, brown) at day 3 postinjury (*n* = 6). S = scab. (B) Graph demonstrating the relative quantification of neutrophil infiltration in the wound at day 3 postinjury (*n* = 6). (C) Immunohistochemistry staining of neutrophils (Ly6G^+^, brown) at day 13 postinjury (*n* = 8). (D) Graph presenting the quantification of neutrophils in the wound at day 13 postinjury (*n* = 8). Magnification = 200× (A) and 400× (C). Scale bar = 50 μm. 0.1% DASA = 0.1% dasatinib ointment, 0.2% DASA = 0.2% dasatinib ointment. Bars indicate the mean ± SEM. Data are analyzed by one-way ANOVA with Tukey’s multiple comparison test. *p < 0.05, **p < 0.01.

Inflammation in the wounds was measured using immunohistochemistry staining. At day 3 postinjury, the results revealed the presence of TNF-α-expressing cells (brown) in the wound areas (Figure 2E). Quantification of TNF-α^+^ cells in the wounds demonstrated that it was significantly reduced following treatment with 0.1 and 0.2% dasatinib ointments (19 ± 6 cells/HPF in 0.1% dasatinib-treated vs 19 ± 5 cells/HPF in 0.2% dasatinib-treated vs 70 ± 6 cells/HPF in control, *p* < 0.05) (Figure 2F). This was confirmed by measuring the TNF-α levels using ELISA (13.9 ± 3.6 pg/mg protein in 0.1% dasatinib-treated vs 14.8 ± 3.2 pg/mg protein in 0.2% dasatinib-treated vs 32.5 ± 4.5 pg/mg protein in control, *p* < 0.01) (Figure 2G). Alongside this observation, the data demonstrated that neutrophil infiltration (Ly6G^+^, brown) was densely packed in the scab in all groups at day 3 postinjury (Figures 3A and S1C), with a reduction in wound neutrophils being observed in both groups of dasatinib ointment-treated mice (Figure 3A). Quantification of the Ly6G^+^ area showed a significant decrease in wound neutrophils in 0.1 and 0.2% dasatinib ointment-treated mice (38 and 63% reduction in Ly6G^+^ area/HPF, respectively, *p* < 0.05), compared to controls at this time point (Figure 3B). At day 13 postinjury, a few neutrophils were detected in the scar (Figure 3C), with no observed alterations in wound neutrophils between all groups (Figure 3D).

**Figure 4:**
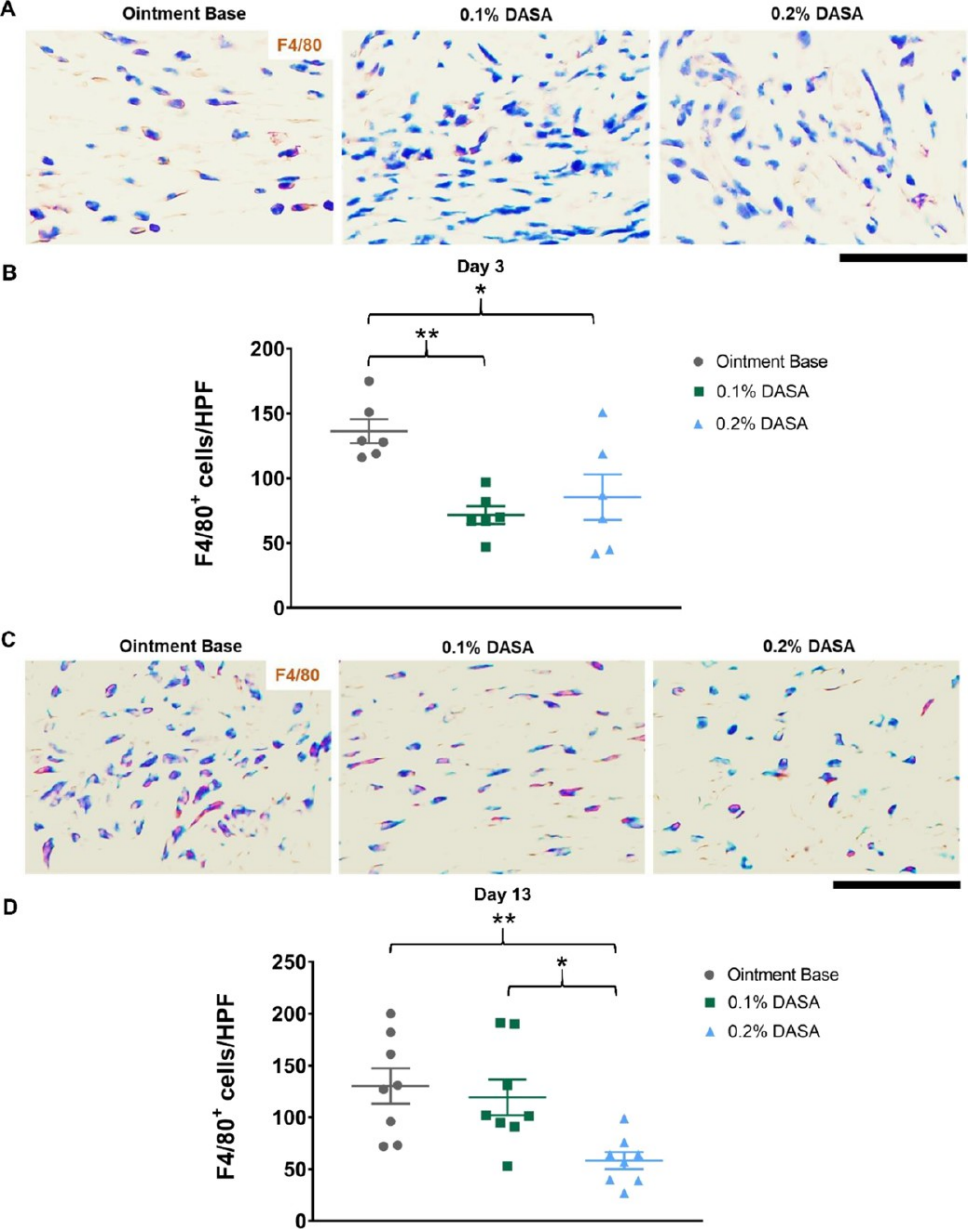
Macrophage infiltration is decreased during the inflammatory phase of repair in dasatinib-treated mice. (A) Immunohistochemistry staining of macrophages (F4/80^+^, brown) at day 3 postinjury (*n* = 6). (B) Graph showing the quantification of macrophage infiltration in the wound at day 3 postinjury (*n* = 6). (C) Immunohistochemistry staining of macrophages (F4/80^+^, brown) at day 13 postinjury (*n* = 8). (D) Graph showing the quantification of macrophages in the wound at day 13 postinjury (*n* = 8). Magnification = 400× (A and C). Scale bar = 50 μm. 0.1% DASA = 0.1% dasatinib ointment, 0.2% DASA = 0.2% dasatinib ointment. Bars indicate the mean ± SEM. Data are analyzed by one-way ANOVA with Tukey’s multiple comparison test. *p < 0.05, **p < 0.01.

**Figure 5:**
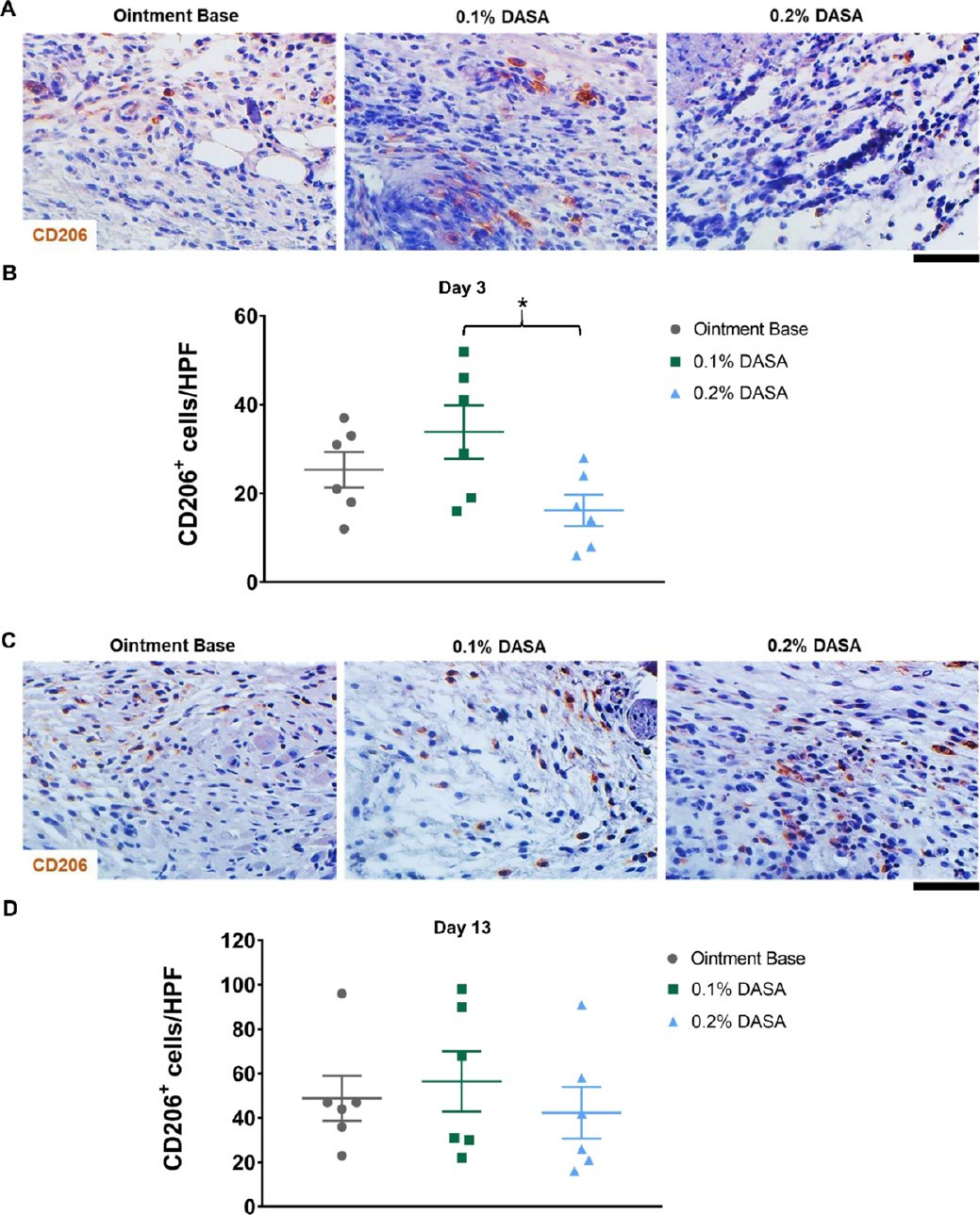
M2 reparative macrophages in the wounds are not impaired following dasatinib treatment. (A) Immunohistochemistry staining of CD206^+^ cells (brown) at day 3 postinjury (*n* = 6). (B) Graph presenting the quantification of CD206^+^ cells in the wound at day 3 postinjury (*n* = 6). (C) Immunohistochemistry staining of CD206^+^ cells (brown) at day 13 postinjury (*n* = 6). (D) Graph demonstrating the quantification of CD206^+^ cells in the wound at day 13 postinjury (*n* = 6). Magnification = 400× (A and C). Scale bar = 50 μm. 0.1% DASA = 0.1% dasatinib ointment, 0.2% DASA = 0.2% dasatinib ointment. Bars indicate the mean ± SEM. Data are analyzed by one-way ANOVA with Tukey’s multiple comparison test. *p < 0.05.

Furthermore, macrophages (F4/80^+^, brown) were detected in the wounds at day 3 postinjury (Figure 4A). Treatment with the dasatinib ointments significantly reduced macrophage infiltration (F4/80^+^ cells) (Figure 4B) relative to the ointment base treatment (72 ± 7 cells/HPF in 0.1% dasatinib-treated vs 86 ± 18 cells/HPF in 0.2% dasatinib-treated vs 136 ± 9 cells/HPF in control, *p* < 0.05). At day 13 postinjury, macrophages remained in the wounds (Figure 4C), and there was no difference in macrophage numbers between 0.1% dasatinib-treated mice and ointment base-treated mice (Figure 4D). Notably, the mice treated with 0.2% dasatinib ointment exhibited a significant decrease in wound macrophages (Figure 4D) compared to the other two groups at day 13 postinjury (58 ± 8 cells/HPF vs 119 ± 17 cells/HPF in 0.1% dasatinib-treated vs 130 ± 17 cells/HPF in control, *p* < 0.05). To further examine the impact of dasatinib ointment on M2 reparative phenotype of macrophages, immunohistochemistry staining of CD206 (a marker of M2 macrophages)^33,34^ was performed. The results showed that a small number of CD206^+^ cells (brown) were present in the wounds at day 3 postinjury (Figure 5A). Mice treated with 0.1% dasatinib ointment demonstrated a comparable number of CD206^+^ cells to the control group (34 ± 6 cells/HPF in 0.1% dasatinib-treated vs 25 ± 4 cells/HPF in control) (Figure 5B). The CD206^+^ cells in the wounds of 0.2% dasatinib-treated mice (16 ± 4 cells/HPF) were significantly lower than 0.1% dasatinib-treated mice (*p* < 0.05), but not the ointment base-treated controls during this early phase (Figure 5B). In the scar at day 13 postinjury, the CD206^+^ cells were also observed (Figure 5C), and there was no difference in CD206^+^ cells between all groups (Figure 5D).

Overall, our results show that the dasatinib ointments produce anti-inflammatory activity by reducing the infiltration of neutrophils and macrophages to the wounds and attenuating TNF-α levels during the early inflammatory phase. With a reduction in wound macrophages, the M2 reparative populations are not impaired following dasatinib treatment.

### Dasatinib Ointment Induces Vascular Leakage and Fibrin(ogen) Deposition during the Inflammatory Phase of Wound Healing

**Figure 6:**
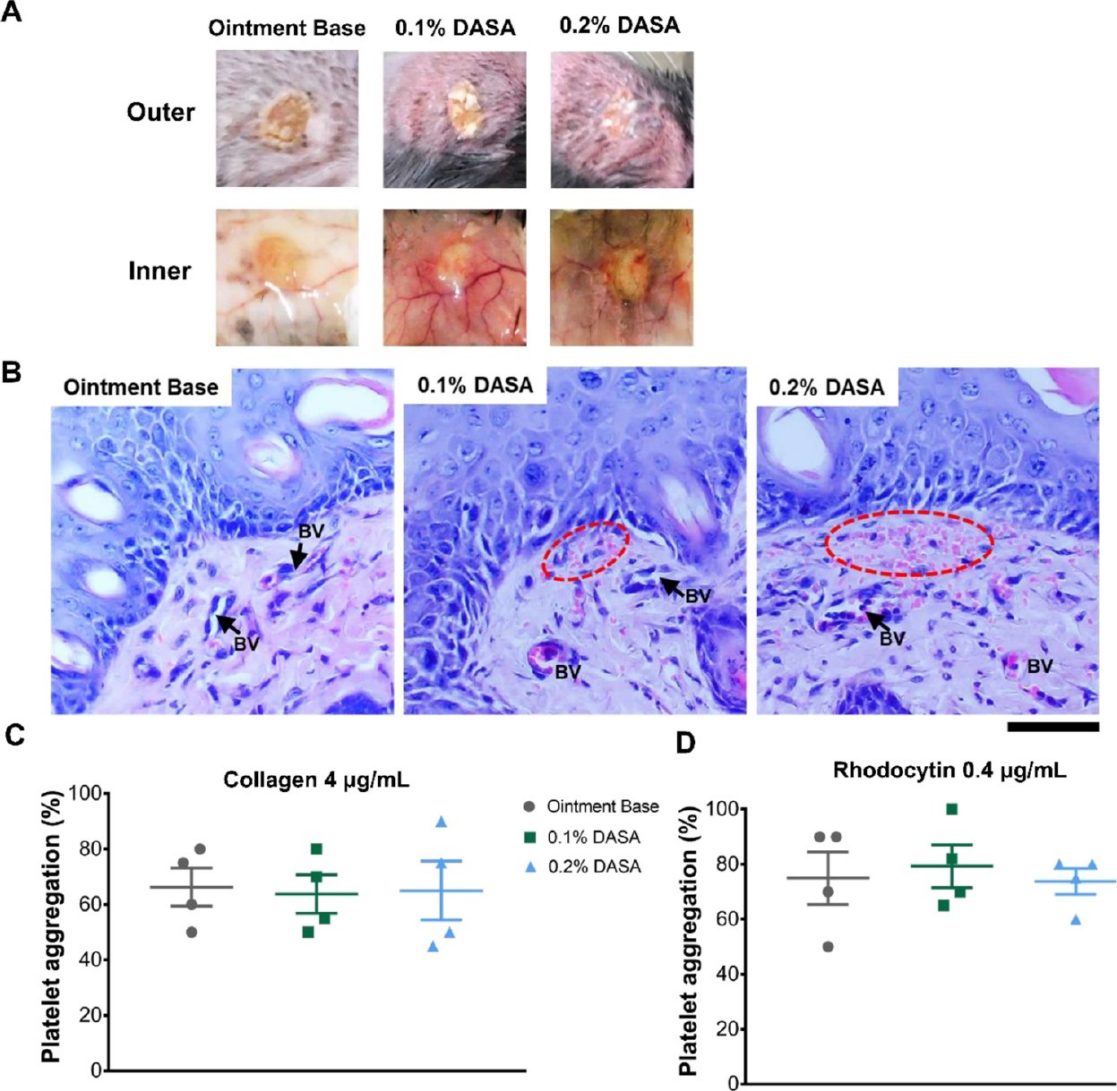
Dasatinib ointment induces vascular leakage during the early phase of wound repair. (A) Wound appearance at day 3 postinjury (*n* = 6). (B) H&E images showing the wound at day 3 postinjury (*n* = 6). The arrows point to blood vessels (BV). The dotted circle indicates extravasated red blood cells. Magnification = 400×. Scale bar = 50 μm. (C) Graph demonstrating ex vivo percent aggregation of platelets (collected at day 3 postinjury) upon stimulation with 4 μg/mL collagen (*n* = 4). (D) Graph showing ex vivo percent aggregation of platelets (collected at day 3 postinjury) upon stimulation with 0.4 μg/mL rhodocytin (*n* = 4).

Given that our previous work reported that dasatinib impaired vascular integrity, in association with fibrin(ogen) deposition during the inflammatory phase to promote the repair process,^5^ we examined whether treatment with the dasatinib ointments would produce this phenotype. Although there was no apparent difference in external appearance of the wounds between all groups at day 3 postinjury (Figure 6A), the inner side of the wounds revealed that the dasatinib ointments induced vascular leakage (Figure 6A) compared to the ointment base-treated wounds. Histological analysis using H&E staining demonstrated that the red blood cells were mainly located in blood vessels in the wounds of the ointment base-treated mice (Figure 6B). In dasatinib ointment-treated mice (both 0.1 and 0.2%), extravasation of red blood cells was detected under the epidermis near the wound edges at day 3 postinjury (Figure 6B). Notably, the ex vivo platelet aggregation following stimulation with collagen (a GPVI ligand) or rhodocytin (a CLEC-2 ligand) was not affected by dasatinib ointment at both day 3 (Figure 6C,D) and day 13 postinjury (Figure S2A,B).

**Figure 7:**
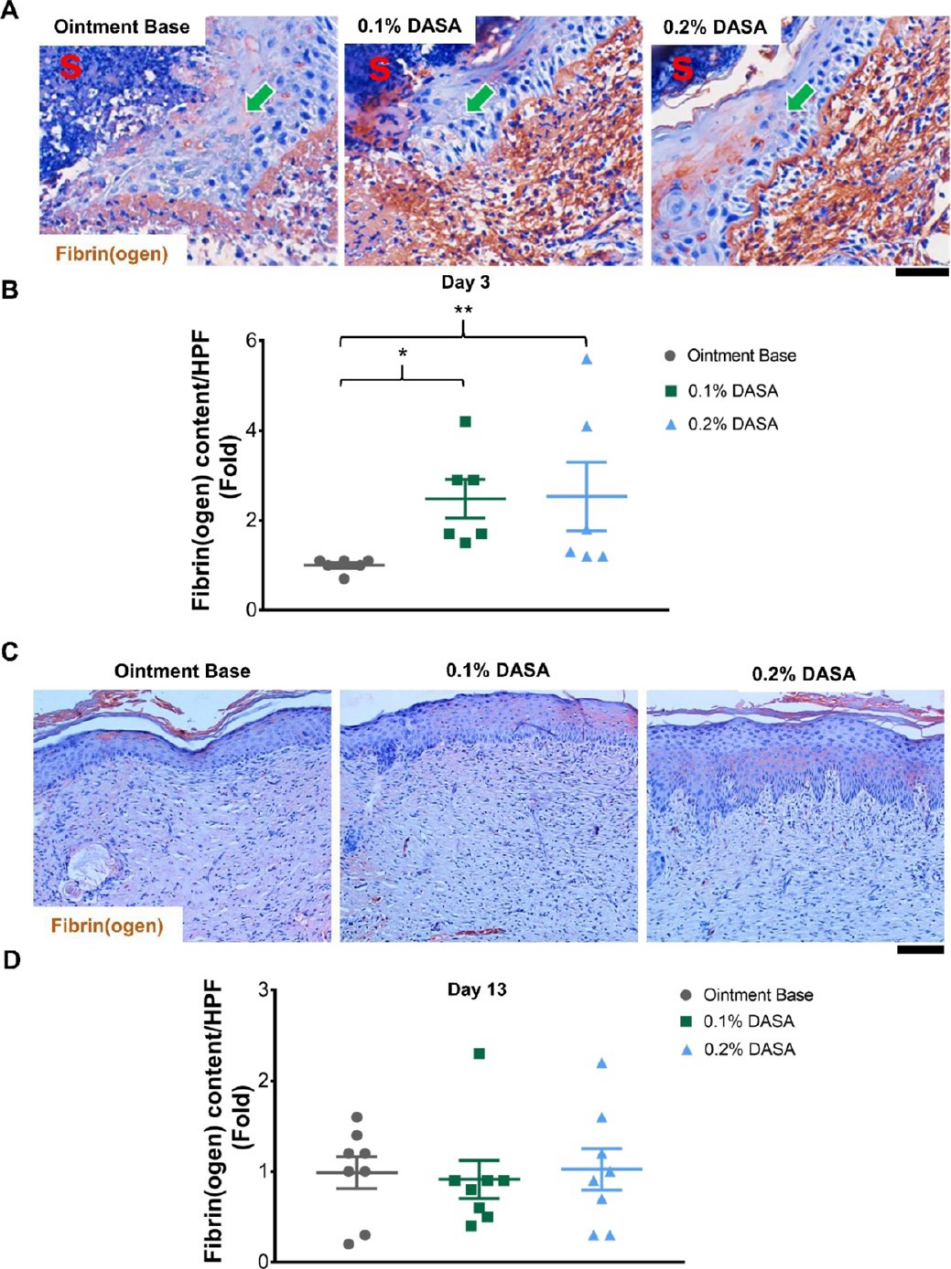
Fibrin(ogen) in the wound is accumulated in the early phase following treatment with dasatinib ointment. (A) Immunohistochemistry staining of fibrin(ogen) (brown) at day 3 postinjury (*n* = 6). Magnification = 400×. Scale bar = 50 μm. The arrows indicate the direction of the epithelial tongue. S = scab. (B) Graph presenting the relative quantification of fibrin(ogen) content in the wound at day 3 postinjury (*n* = 6). (C) Immunohistochemistry staining of fibrin(ogen) (brown) at day 13 postinjury (*n* = 8). Magnification = 200×. Scale bar = 100 μm. (D) Graph demonstrating the relative quantification of fibrin(ogen) content at day 13 postinjury (*n* = 8). 0.1% DASA = 0.1% dasatinib ointment, 0.2% DASA = 0.2% dasatinib ointment. Bars indicate the mean ± SEM. Data are analyzed by Kruskal-Wallis test with Dunn’s multiple comparison test. *p < 0.05, **p < 0.01.

The fibrin(ogen) content in the wound was measured using immunohistochemistry. In normal skin, fibrin(ogen) was detected mainly in blood vessels (Figure S3A,B). During day 3 postinjury, the results showed that fibrin(ogen) was present in the scab (Figure S3C) and underneath the epithelial tongues (Figure 7A). Quantification of the fibrin(ogen) content demonstrated a significant increase in fibrinogen deposition in the wounds following treatment with the dasatinib ointments (2.5-fold increase in fibrin(ogen)^+^ signal/HPF in both 0.1 and 0.2% dasatinib-treated, *p* < 0.05) relative to the ointment base control group at day 3 postinjury (Figure 7B). At day 13 postinjury, only a minor level of fibrin(ogen) was found in the upper part of the scar (Figure 7C), and there was no difference in fibrin(ogen) content between all groups (Figure 7D).

These results demonstrate that dasatinib ointment induces vascular leakage and fibrin(ogen) deposition in the wounds during the inflammatory phase, which potentially facilitates the repair process. The accumulated fibrin(ogen) was removed in the later phase. Moreover, topical dasatinib might be minimally absorbed into the systemic circulation, given that the ointments did not significantly inhibit ex vivo platelet aggregation upon GPVI and CLEC-2 activation during the course of wound healing, suggesting a low risk of systemic side effects.

## Discussion

In the present study, we show the therapeutic potential of dasatinib ointment in facilitating the healing of a full-thickness excisional skin wound in mice. The data demonstrated that 0.1% dasatinib ointment accelerated skin wound healing in association with reduced inflammation, impaired vascular integrity, increased fibrin(ogen) accumulation, improved keratinocyte proliferation, and enhanced angiogenesis at an early phase without affecting the late phase of repair in mice. However, 0.2% dasatinib ointment induced minor wound bleeding and scab reformation during the late phase, which contributed to delayed healing, although it appeared to promote the early phase of repair.

Inflammation has been shown to impair wound healing.^4,35,36^ Chronic or dysregulated inflammation is a major contributing factor for non-healing wounds in diabetic ulcers^32,35,36^ and chronic venous ulcers.^4,37^ In animal models of wound injury, a reduction in inflammation due to neutrophil depletion has been reported to accelerate healing.^38,39^ It is suggested that neutrophils release various tissue-damaging mediators, including enzymes (e.g., proteases, elastases, and myeloperoxidases),^38,39^ reactive oxygen species (ROS),^38,39^ and neutrophil extracellular traps,^40^ which potentially delay wound healing. Macrophages have also been demonstrated to contribute to non-healing wounds by promoting chronic inflammation.^41,42^ In addition, the depletion of macrophages at an early stage displayed a reduction in scar size at the end.^43^ Therefore, in our study, the anti-inflammatory activity of dasatinib ointments, via the attenuated infiltration of neutrophils and macrophages and the reduction of TNF-α levels in wounds during the early phase, may contribute to facilitating wound repair. In agreement with our data, previous in vivo studies have revealed that oral and parenteral dasatinib reduces inflammation in animal models of lung^18,19^ and skin inflammation,^24^ including during the skin wound healing observed in our previous work.^5^ In addition, dasatinib showed direct inhibition of neutrophil functions.^44^ More importantly, 0.02–0.1% topical dasatinib solutions, comparable concentrations to our study, have been shown to attenuate inflammation in an animal model of atopic dermatitis,^24^ supporting the potential anti-inflammatory activity of dasatinib ointment.

Given that a deficiency in M2 reparative macrophages contributes to chronic inflammation and impaired wound healing, including in diabetic wound,^33,45^ interventions that promote M2 polarization of macrophages may improve wound healing.^33,46^ With a decrease in macrophage infiltration to the wound at early phase following treatment with dasatinib ointments, our data showed that the numbers of CD206^+^ cells were comparable to untreated mice, suggesting that the majority of wound macrophages in dasatinib-treated groups, particularly 0.1% dasatinib, might be M2 populations. The prohealing phenotype of M2 macrophages^34,45,47^ might also support the reduced inflammation and enhanced angiogenesis during dasatinib treatment. In agreement with our observations, a previous study has reported that macrophage depletion did not affect the early phase of wound healing, but led to defective angiogenesis and delayed healing in the later phase due to a lack of M2 macrophages.^43^

Impaired vascular integrity might promote wound healing by enhancing the extravasation of repair-associated molecules, including fibrinogen and growth factors.^5−8^ In addition, due to a small hole generated during leukocyte diapedesis (approximately 4 μm in width and 6 μm in length),^12^ extravasation of red blood cells (5–6 μm in diameter)^14^ was detected during inflammatory bleeding.^11,13^ However, there is no evidence demonstrating an accumulation of extravasated leukocytes in this setting,^11,13^ possibly because of their larger size (∼12–16 μm in diameter for blood neutrophils and monocytes).^15^ Fibrin(ogen) plays multiple roles to facilitate wound healing, including promoting the migration of keratinocytes and endothelial cells, contributing to re-epithelialization and angiogenesis.^5,6^ In addition, fibrin(ogen) may inhibit the infiltration of neutrophils and macrophages into wounds.^5,6^ Therefore, vascular leakage and fibrin(ogen) deposition in wounds during the inflammatory phase following treatment with dasatinib ointment, particularly 0.1% dasatinib, may contribute to the accelerated healing.

Regarding the potential mechanisms underlying dasatinib-induced vascular leakage, the data at day 3 postinjury in our previous study^5^ showed a dasatinib plasma level of 15 ng/mL (30 nM) at 1 h following 5 mg/kg dasatinib i.p. injection, a dose that promoted wound healing in mice (data not shown). At this time point, platelet aggregation upon activation of GPVI and CLEC-2 was inhibited in association with intrawound bleeding.^5^ Similar results of wound healing were observed in GPVI and CLEC-2 double knockout mice.^6^ However, other studies have shown that dasatinib directly disrupted the endothelial junction, possibly by ROS production^48^ or activation of the Rho-kinase (ROCK)/myosin light chain (MLC) signaling pathway^49^ to impair vascular integrity, but the dose of dasatinib was relatively high (≥10–70 mg/kg i.p. injection in vivo and ≥100 nM in vitro).^17,48,49^ Therefore, following treatment with the low concentration dasatinib ointments in the present study, it might be possible that the local inhibition of platelet functions plays a primary role in the induction of vascular hyperpermeability at the early phase of repair^5,6^ although determining real-time alterations of platelet activity at the wound vasculatures using intravital microscopy^12^ might provide a clear mechanistic insight into the effect of dasatinib ointment on vascular integrity.

Nonetheless, minor wound bleeding was observed at the late phase of repair (day 9 postinjury) in the 0.2% dasatinib ointment-treated group, which resulted in scab reformation and delayed healing at the end. This side effect might be due to a loss of integrity of newly synthesized blood vessels in the wounds. In addition to the direct effect of dasatinib on vascular integrity (through the inhibition of platelet functions^5,6^ or disruption of endothelial junctions^17,48,49^), we proposed that a reduction in macrophages in the later phase might contribute to wound bleeding, given that we observed a significant decrease in wound macrophages at day 13 postinjury in the 0.2% dasatinib ointment-treated group relative to animals treated with 0.1% dasatinib ointment and ointment base. In agreement with this, a previous study demonstrated that the depletion of macrophages at the tissue maturation phase (day 4 to day 9 postinjury) resulted in wound bleeding due to an increase in endothelial cell damage and apoptosis, suggesting that macrophages play a role in promoting endothelial integrity during this time.^43^ Although alteration in peripheral blood monocytes was not determined, a reduction in wound macrophages on day 13 postinjury following treatment with 0.2% dasatinib ointment was unlikely to be due to myelosuppression, given that we did not observe impaired function of circulating platelets during the course of wound healing. In addition, common characteristics of dasatinib-induced cytopenia include neutropenia, thrombocytopenia, and anemia, but not monocytopenia.^50−52^ To minimize the unwanted minor wound bleeding in the late phase of repair, it is interesting to investigate whether a lower concentration of dasatinib ointment (e.g., 0.02–0.05%)^24^ would benefit in wound healing, particularly in a model of diabetic^35,36^ or venous ulcers,^4,37^ where chronic inflammation and ischemia play a significant detrimental role in non-healing wounds.

The increased risk of wound infections is a concern following a reduction in inflammatory cells. In a single case report of an acute lymphoblastic leukemia patient who took long-term dasatinib tablets (100 mg daily for three cycles), surgical wound infection and delayed healing developed following a total knee replacement. As a result, intermittent dose reductions (dasatinib 100 mg every other day for several weeks) were selected in the later cycles of cancer treatment, together with antimicrobial therapy to promote wound healing.^53^ In fact, the peak plasma concentration following the oral intake of 100 mg dasatinib in humans is approximately 43.8–112.4 ng/mL,^54^ which is relatively higher than the concentration observed in our wound healing studies (plasma dasatinib level in mice = 15 ng/mL).^5^ In addition, it has been previously shown that 12 ng/mL of dasatinib is capable of inhibiting Src in human and murine bone marrow-derived mononuclear cells in vitro.^55^ In agreement with this, a positive impact of the anti-inflammatory activity of dasatinib in vivo could be seen at a dose as low as 1 mg/kg oral administration^18,19^ or ≤0.1% topical use,^24^ supporting that short-term, low concentrations (0.1–0.2%) of dasatinib ointment might not increase susceptibility to infections. In addition, mice in the present study were housed in a cleaned conventional cage after biopsy and until the end of experiment without exhibiting any signs and symptoms of wound infection (e.g., erythema and purulent exudate) although the wound took 14 days to completely close, relative to 10 days in our previous work,^5,6^ where animals were kept in an individually ventilated cage to prevent contaminations. These data indicate that wound infections due to the anti-inflammatory activity of dasatinib ointment are unlikely to occur in proper wound care settings. However, the impact of dasatinib ointment (alone or in combination with an antimicrobial agent) on the healing of infected wounds^56^ requires further investigations.

In conclusion, we describe that 0.1% dasatinib ointment facilitates wound healing in a murine model of excisional skin wound by reducing inflammation and promoting vascular leakage-mediated fibrin(ogen) deposition, in association with increased keratinocyte proliferation, enhanced angiogenesis, and rapid reduction in wound size at the early phase, without affecting the late phase of repair. Although 0.2% dasatinib ointment promotes the early phase of repair, it induces minor wound bleeding and scab reformation during the late phase, which contribute to delayed healing. Therefore, a low-dose dasatinib ointment provides a positive impact on wound healing. The anti-inflammatory, vascular hyperpermeability, and angiogenic activities of dasatinib ointment might represent an interesting property to further investigate its role in settings where inflammation and ischemia predominantly contribute to non-healing wounds, including diabetic and venous ulcers.

## Supporting Information Available

Additional immunohistochemistry data and platelet aggregation analysis (PDF)
