# Supplementary material for: Dasatinib Ointment Promotes Healing of Murine Excisional Skin Wound
Source: ACS Pharmacol Transl Sci. 2023 Jun 9;6(7):1015–27. doi: 10.1021/acsptsci.2c00245 (PMC10353058; doi:10.1021/acsptsci.2c00245)
Supplement: Supplementary file 1 — pt2c00245_si_001.pdf [file pt2c00245_si_001.pdf]

Supporting Information

Dasatinib ointment promotes healing of murine excisional skin wound

Surasak Wichaiyo[1, 2,*], Saovaros Svasti[3, 4], Arnatchai Maiuthed[1, 2], Pattarawit Rukthong[5], Arman Syah Goli[1] and Noppawan Phumala Morales[6]

[1]Department of Pharmacology, Faculty of Pharmacy, Mahidol University, Bangkok, Thailand

[2]Centre of Biopharmaceutical Science for Healthy Ageing, Faculty of Pharmacy, Mahidol University, Bangkok, Thailand

[3]Thalassemia Research Center, Institute of Molecular Biosciences, Mahidol University, Nakhon Pathom, Thailand

[4]Department of Biochemistry, Faculty of Science, Mahidol University, Bangkok, Thailand

[5]Department of Pharmaceutical Technology, Faculty of Pharmacy, Srinakharinwirot University, Nakhonnayok, Thailand

[6]Department of Pharmacology, Faculty of Science, Mahidol University, Bangkok, Thailand

***Correspondence:** Surasak Wichaiyo, PhD

Department of Pharmacology, Faculty of Pharmacy, Mahidol University,

447 Sri-Ayuthaya, Rajathevi, Bangkok, Thailand, 10400

Email: surasak.wic@mahidol.ac.th, Tel & Fax: +66 0-2644-8700

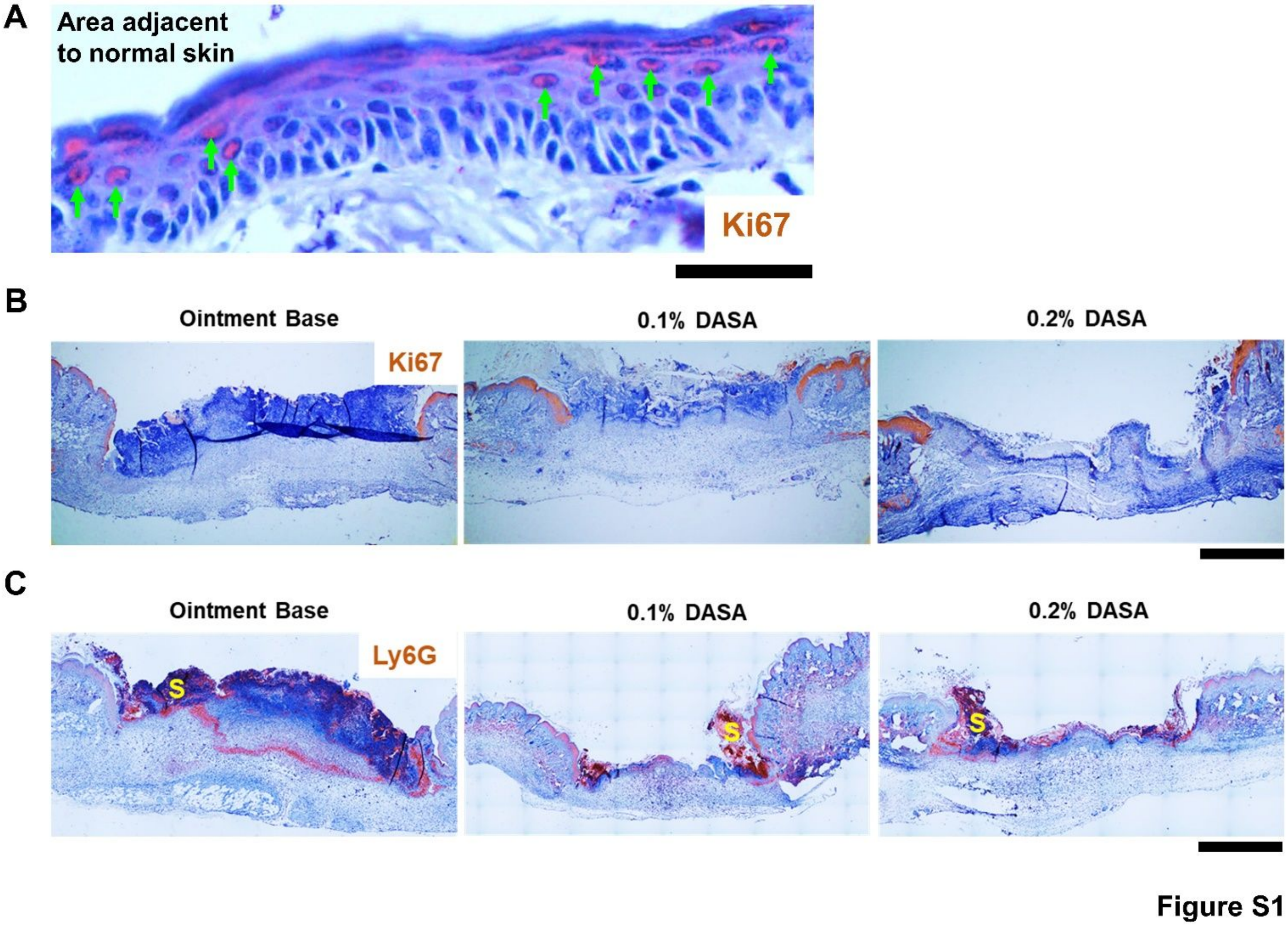

Figure S1. Additional immunohistochemistry data of Ki67 and Ly6G**.** (A) Ki67 staining showing positive signal (brown color) in the nucleus of keratinocytes (arrows) at the area adjacent to normal skin. (B) Representative images of Ki67 staining in the entire wound at day 3 postinjury. (C) Representative images of Ly6G staining in the entire wound at day 3 postinjury. Magnification (A: 400X and B, C: 40X). Scale bar (A: 50 µm and B, C: 500 µm).

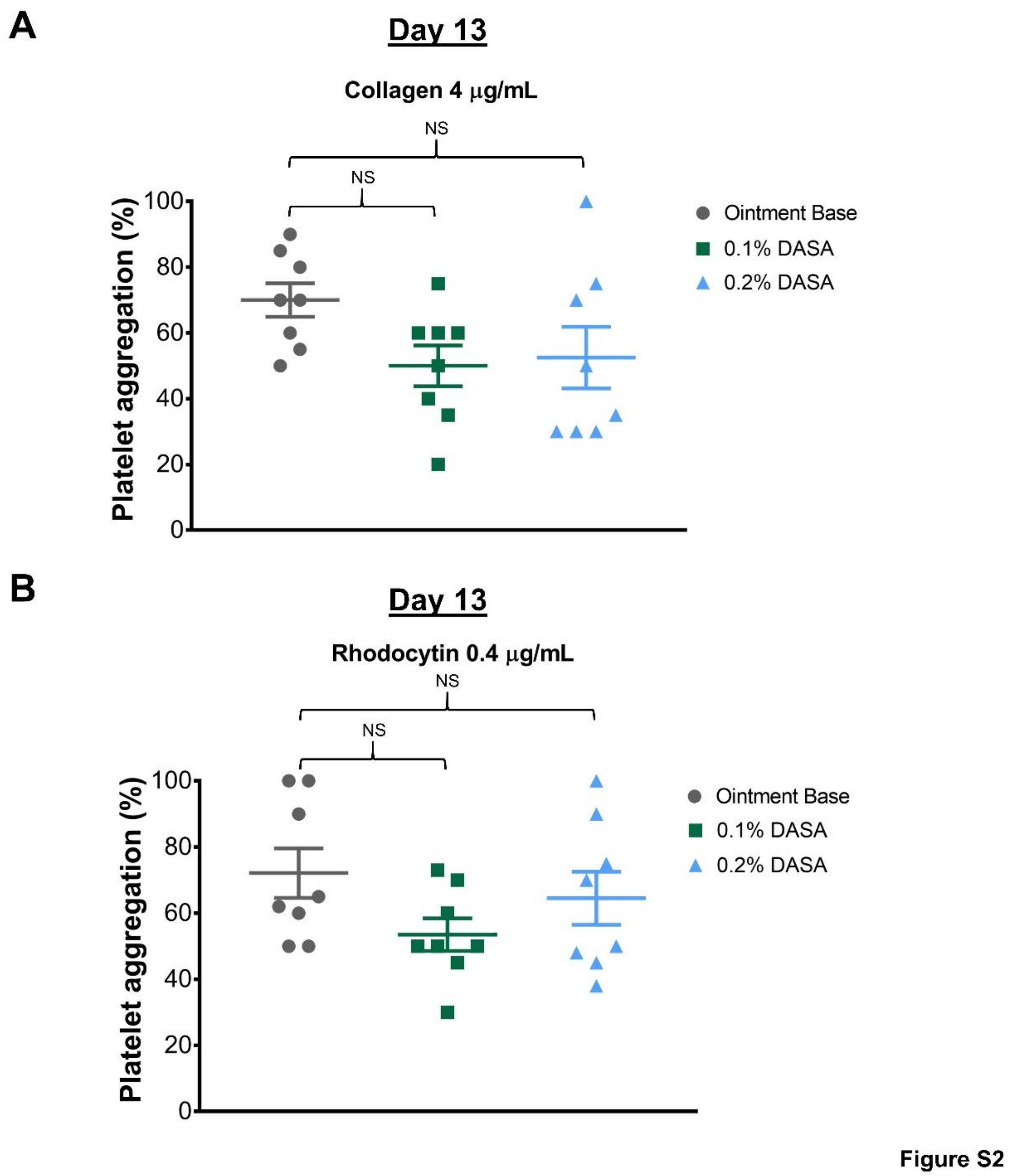

Figure S2. Platelet aggregation data on day 13 postinjury. (A) Graph demonstrating *ex vivo* percent aggregation of platelets (collected at day 13 postinjury) upon stimulation with 4 μg/mL collagen (n=8). (B) Graph showing *ex vivo* percent aggregation of platelets (collected at day 13 postinjury) upon stimulation with 0.4 μg/mL rhodocytin (n=8). NS = not statistically significant.

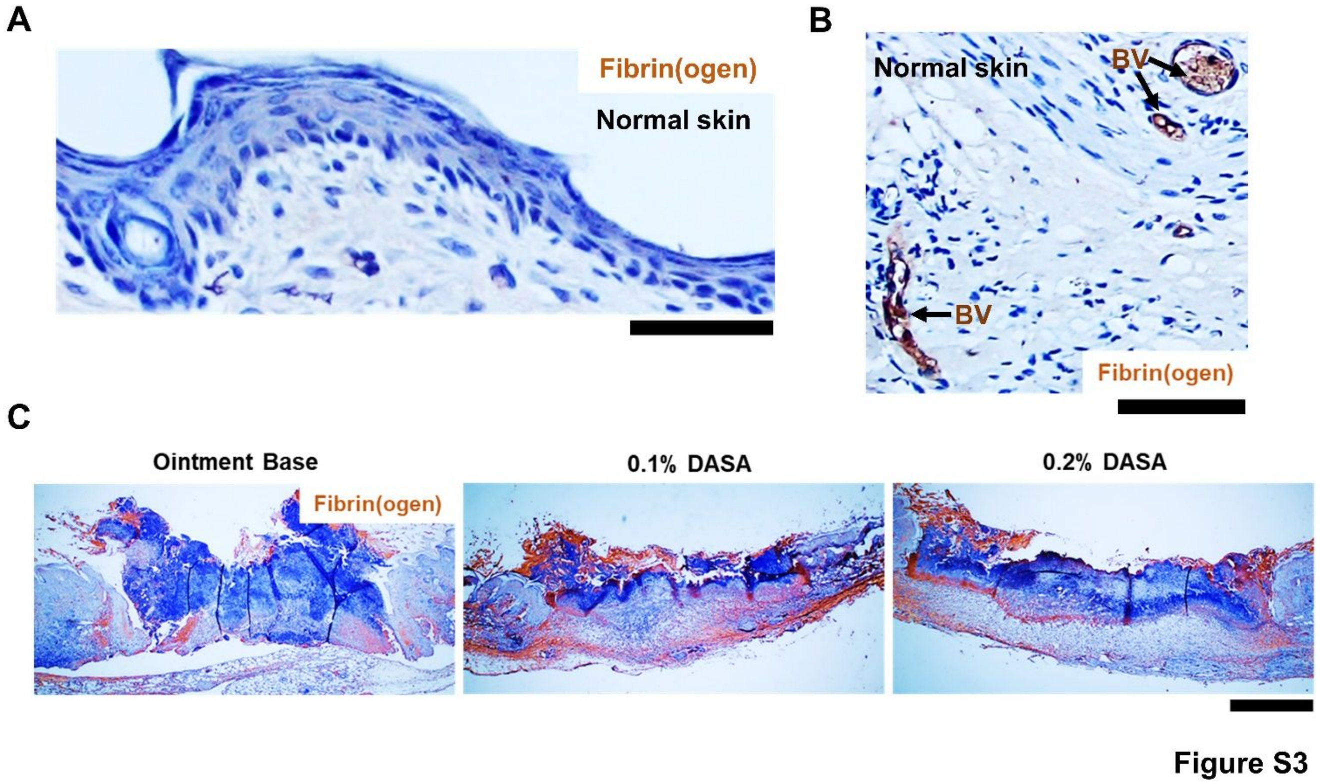

Figure S3. Additional immunohistochemistry data of fibrin(ogen). (A-B) Fibrin(ogen) staining of normal skin as negative control. BV = blood vessels. (C) Representative images of fibrin(ogen) staining in the entire wound at day 3 postinjury. Magnification (A: 400X, B: 200X and C: 40X). Scale bar (A: 50 µm, B: 100 µm and C: 500 µm).
